# Evaluation of a Rapid Antigen Test To Detect SARS-CoV-2 Infection and Identify Potentially Infectious Individuals

**DOI:** 10.1128/JCM.00896-21

**Published:** 2021-08-18

**Authors:** Michael Korenkov, Nareshkumar Poopalasingam, Matthias Madler, Kanika Vanshylla, Ralf Eggeling, Maike Wirtz, Irina Fish, Felix Dewald, Lutz Gieselmann, Clara Lehmann, Gerd Fätkenheuer, Henning Gruell, Nico Pfeifer, Eva Heger, Florian Klein

**Affiliations:** a Institute of Virology, Faculty of Medicine and University Hospital Cologne, University of Cologne, Cologne, Germany; b Methods in Medical Informatics, Department of Computer Science, University of Tübingen, Tübingen, Germany; c German Center for Infection Research, Partner Site Bonn-Cologne, Cologne, Germany; d Department I of Internal Medicine, Faculty of Medicine and University Hospital Cologne, University of Cologne, Cologne, Germany; e Center for Molecular Medicine Cologne, University of Cologne, Cologne, Germany; f Faculty of Medicine, University of Tübingen, Tübingen, Germany; g German Center for Infection Research, Partner Site Tübingen, Tübingen, Germany; Rhode Island Hospital

**Keywords:** SARS-CoV-2, antigen test, RT-qPCR, virus culture, infectiousness

## Abstract

The identification and isolation of highly infectious SARS-CoV-2-infected individuals is an important public health strategy. Rapid antigen detection tests (RADT) are promising tools for large-scale screenings due to timely results and feasibility for on-site testing. Nonetheless, the diagnostic performance of RADT in detecting infectious individuals is not yet fully determined. In this study, RT-qPCR and virus culture of RT-qPCR-positive samples were used to evaluate and compare the performance of the Standard Q COVID-19 Ag test in detecting SARS-CoV-2-infected and possibly infectious individuals. To this end, two combined oro- and nasopharyngeal swabs were collected at a routine SARS-CoV-2 diagnostic center. A total of 2,028 samples were tested, and 118 virus cultures were inoculated. SARS-CoV-2 infection was detected in 210 samples by RT-qPCR, representing a positive rate of 10.36%. The Standard Q COVID-19 Ag test yielded a positive result in 92 (4.54%) samples resulting in an overall sensitivity and specificity of 42.86 and 99.89%, respectively. For adjusted *C_T_* values of <20 (*n* = 14), <25 (*n* = 57), and <30 (*n* = 88), the RADT reached sensitivities of 100, 98.25, and 88.64%, respectively. All 29 culture-positive samples were detected by the RADT. Although the overall sensitivity was low, the Standard Q COVID-19 Ag test reliably detected patients with high RNA loads. In addition, negative RADT results fully corresponded with the lack of viral cultivability in Vero E6 cells. These results indicate that RADT can be a valuable tool for the detection of individuals with high RNA loads that are likely to transmit SARS-CoV-2.

## INTRODUCTION

Timely diagnosis of SARS-CoV-2 infection with subsequent contact tracing and rapid isolation is a critical public health strategy to contain the COVID-19 pandemic (1–3). The current gold standard of SARS-CoV-2 testing is based on real-time reverse transcription-PCR (RT-qPCR) (4). However, due to higher costs, longer turnaround times, and higher sensitivities, RT-qPCR is less suited for rapid point-of-care identification of infectious individuals, since it is also able to detect nonreplicating viruses (5–7). Therefore, there is a need for an inexpensive alternative testing method to directly detect infectious individuals that can be deployed widely without the use of specialized equipment (8, 9).

One promising approach is the use of lateral flow immunochromatographic assays, commonly referred to as rapid antigen detection tests (RADT), designed to detect viral antigens. RADT are of particular use for community-based screenings due to low turnaround times and feasibility for on-site testing (10, 11). Numerous tests have already been approved for clinical use; however, performance studies under real-life conditions evaluating the quality of RADT using not only RT-qPCR but also virus cultures as a reference for infectivity are limited (12, 13). In published studies, reported test characteristics, such as sensitivity, varied greatly depending on cohort composition (24.3 to 89%) (14–26).

As high RNA loads are typically associated with a higher probability of infectiousness, the diagnostic performance of RADT in the context of infectivity models is yet to be determined (27–29). Therefore, there is a need for large-scale field studies with a focus on virus cultivability to be able to appropriately interpret RADT results. Here, we examined the performance of RADT in detecting both infected and infectious individuals in a routine diagnostic center using RT-qPCR and virus culturing as reference methods.

## MATERIALS AND METHODS

### Study design.

Between 26 October 2020 and 11 January 2021, all individuals were tested for SARS-CoV-2 infection by RT-qPCR at the University Hospital Cologne, Department I of Internal Medicine, Cologne, Germany. SARS-CoV-2 testing was available for individuals from the general population with COVID-19 symptoms, suspected disease, or SARS-CoV-2 exposure as part of routine diagnostics, as well as for hospital staff members as part of screening measures. For quality control, an RADT was simultaneously performed after verbal consent. Two swab specimens, each collected from oro- and nasopharynges, were obtained by the same trained personnel. The first swab was transferred into virus transport and preservation medium (Biocomma, Shenzhen, China) or BD ESwab (Becton Dickinson, Franklin Lakes, NJ, USA) for RT-qPCR testing, and the second swab was used for on-site RADT testing. All samples for RT-qPCR were routinely processed within 12 h after collection. Upon approval by the Institutional Review Board of the University of Cologne, results were retrospectively analyzed, including clinical data retrieved from a symptoms diary webtool that all individuals registering for a SARS-CoV-2 test are asked to complete. Due to several implausible self-reported entries, only webtool entries not older than a week from the time of testing were included into symptom analysis. Patients were categorized as symptomatic if the reported symptom duration at the time of testing was ≤14 days and one of the following symptoms was found: fever, cough, rhinorrhea, nausea, diarrhea, shortness of breath, and/or a new olfactory or taste disorder.

### Rapid antigen detection test.

The Standard Q COVID-19 Ag test (SD Biosensor, Inc., Suwon-si, Republic of Korea/Hoffmann La Roche AG, Basel, Switzerland) is a rapid chromatographic immunoassay for the qualitative detection of SARS-CoV-2 nucleocapsid protein. The test was performed according to the manufacturer’s instructions using the enclosed dry swab for sample collection with one modification. Instead of a nasopharyngeal swab only, a combined oro- and nasopharyngeal swab was performed to ensure comparability with RT-qPCR sample collection. The operating instructions, in brief, were as follows. The collected swab was mixed in the provided tube of collection medium, and three drops were applied through a nozzle cap onto the test strip. Results were read out visually after 15 to 20 min by medically trained and instructed personnel. In accordance with the manufacturer’s reference guide, faint lines were considered positive if the control line was also present.

### Real-time reverse transcription-PCR.

RT-qPCR was performed using different SARS-CoV-2 RNA detection protocols that were normalized according to the same standard. The following SARS-CoV-2 detection protocols were utilized. (i) Nucleic acid extraction was done for 935 (46.10%) samples using the MagNA Pure 96 system DNA and viral NA large volume kit (Roche Diagnostics, Mannheim, Germany) according to the manufacturer’s instructions. After RNA purification from 500 μl of viral transport medium and elution into 100 μl of elution buffer, multiplex RT-qPCR was performed using an in-house N-gene primer set on a LightCycler 480 II system (Roche Diagnostics). The LightMix SarbecoV E-gene plus equine arteritis virus (EAV) control kit (TIB Molbiol, Berlin, Germany) was included in every RT-qPCR run. (ii) A cobas SARS-CoV-2 test kit running on the cobas 6800 (Roche Diagnostics) was used for 407 (20.07%) samples targeting the viral E-gen and ORF1a/b regions according to the manufacturer’s instructions. (iii) A SARS-CoV-2 AMP kit running on an Alinity m (Abbott, Chicago, IL, USA) was used for 63 (3.11%) specimens targeting the viral N and RdRp genes according to the manufacturer’s instructions. (iv) A multiplex RT-qPCR with a LightMix SarbecoV E-gene (TIB Microbiol), an in-house N-gene primer/probe set, and a human β-globin primer set as an internal control, running on the Panther Fusion (Hologic, Wiesbaden, Germany), was used for 36 (1.78%) samples. (v) For 579 (28.55%) specimens, samples from up to 10 asymptomatic employees were pooled and tested for SARS-CoV-2 using the methods described in steps i, ii, and iii. Positive pools were resolved, and samples were tested separately as described previously in steps i to iv. (vi) An Xpert Xpress SARS-CoV-2 (Cepheid, Sunnyvale, CA, USA) test kit was used for 8 (0.39%) samples according to the manufacturer’s instructions.

To enable comparison of cycle threshold (*C_T_*) values obtained by the different RT-qPCR methods, *C_T_* values were translated into copies/ml and then converted to a cobas 6800 adjusted *C_T_* value. For this purpose, seven serial dilutions from a high titer SARS-CoV-2 sample were tested by all five RT-qPCR methods described above. Standard curves for each amplification method were generated by a regression model. For the following conversion of device-specific *C_T_* values into copies/ml, two SARS-CoV-2 samples with a quantified RNA load from INSTAND (Society for the Promotion of Quality Assurance in Medical Laboratories, e.V., Düsseldorf, Germany; in cooperation with the Robert Koch-Institute and the Institute of Virology, Charité, Berlin) were tested on every device and subsequently used for *C_T_*-based absolute RNA quantification.

### SARS-CoV-2 culture.

Vero E6 cells (ATCC CRL-1586) were cultured in complete medium (CM) consisting of Dulbecco modified Eagle medium (Thermo Fisher Scientific/Gibco, Waltham, MA, USA) supplemented with 10% fetal calf serum (FCS; GE Healthcare, Chicago, IL, USA), 200 U/ml penicillin, 200 μg/ml streptomycin, 0.25 μg/ml amphotericin B, 2 mM l-glutamine, and 1 mM sodium pyruvate (all from Thermo Fisher Scientific/Gibco) at 37°C in an incubator with 5% CO_2_. One day prior to infection, 3 × 10^5^ cells were seeded onto T25 flasks in 5 ml of CM. Retained swab samples were stored for up to 8 h at 4°C in transport medium until RT-qPCR results became available. For virus cultures, 250 μl of SARS-CoV-2 RT-qPCR-positive samples were diluted 1:5 in infection medium (IM) consisting of complete medium with FCS reduced to 2%. After removal of the cell culture supernatant, the diluted samples were added to Vero E6 cells and incubated for 1 h at 37°C and 5% CO_2_. After washing with 5 ml of phosphate-buffered saline (Thermo Fisher Scientific/Gibco), 5 ml of IM was added, and the cells were cultured for 7 days at 37°C and 5% CO_2_. The cells were checked for the presence of cytopathic effects (CPE) on days 4 and 7. On both days, 1 ml of culture supernatant was harvested and stored at −80°C for conformation of positive cultures through RT-qPCR as described above. An increase in RNA load and unchanged RNA loads of >9 log_10_ copies/ml between day 4 and day 7 were considered successful virus cultivation. All virus isolation experiments were performed under BSL-3 conditions.

### Dilution series.

To validate our SARS-CoV-2 culturing approach, four dilution series with sample CT values ranging from 15.5 to 17.6 were analyzed for virus recovery and RADT result. Two hundred fifty microliters of retained samples was thawed and serially diluted in IM (1:5). Virus cultivation and RT-qPCR were performed as described above. RADT were performed with 250 μl of original samples and each dilution as described above. Virus cultivation and RADT testing showed that all SARS-CoV-2-positive cultures were previously detected by RADT (see Fig. S1 in the supplemental material).

### Statistical analysis.

Sensitivity (positive percent agreement) and specificity (negative percent agreement), as well as positive and negative predictive values, were calculated using RT-qPCR as a reference. Culture and RADT results were evaluated by a contingency table, and a *P* value was calculated with Fisher exact test. Confidence intervals (CI) were calculated using the Wilson/Brown method. Mann-Whitney U-test (MWU) was used to compare differences between medians. *P* values of <0.05 were considered significant. Probit regression was carried out using a generalized linear model (R-function glm) with the probit link function. To correct for repeated measurements from the same individual on different days, the basic analyses were modified as follows. (i) Confusion matrices for the calculation of all performance measures were calculated in a weighted manner so that the contribution of a single test is inversely proportional to the number of tests taken from the corresponding individual. (ii) To compare two populations of data points, a generalized estimating equation (R-function gee::gee) was fitted using each patient as its own cluster and an exchangeable correlation structure. (iii) Probit regression was carried out by fitting a generalized linear mixed model with probit link function and random intercepts for each individual (R-function GLMMadaptive::mixed_model). Marginal means and confidence bands were calculated with R-function ggeffects::ggpredict. Data analysis was performed using Microsoft Excel 16.44 (Microsoft), Prism 9 (GraphPad Software, Inc.), Python 3.8.3, and R 3.6.3.

### Ethics.

The Institutional Review Board of the University of Cologne acknowledged and approved the study under application 21-1039.

### Data availability.

All data used in the analysis will be made available upon request.

## RESULTS

### RT-qPCR and RADT testing in a large cohort under real-life conditions.

**FIG 1:**
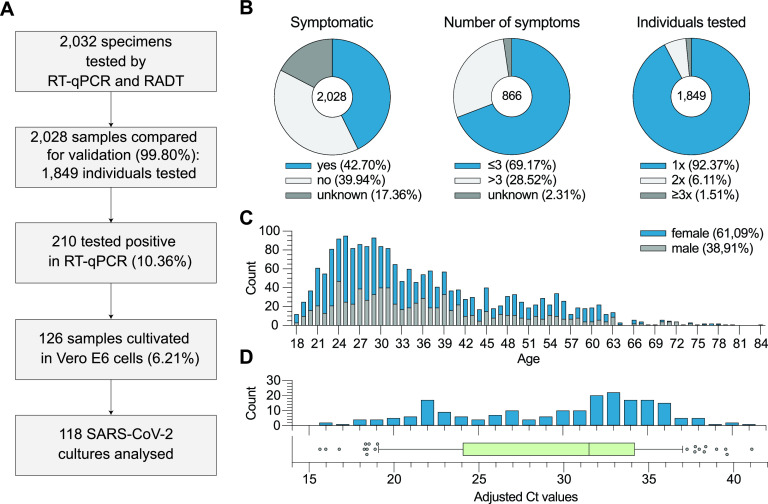
Study procedure and cohort description. (A) Flowchart including cohort sizes selected for RT-qPCR, RADT, and cell culture assays. Percentages in boxes 3 and 4 refer to all analyzed samples (*n* = 2,028). (B) Distribution and number of symptoms, as well as test frequencies, among individuals. (C) Age and gender distribution of the cohort. (D) Distribution of cycle threshold (*C_T_*) values (adjusted to cobas 6800).

To validate RADT performance, two swabs, each obtained from oro- and nasopharynges, were collected and tested using both RT-qPCR and RADT. RT-qPCR-positive samples were additionally cultivated in Vero E6 cells to determine the ability of the RADT to detect replication-competent virus in individual specimens. A total of 2,032 samples were tested, 4 of which were excluded due to 3 RADT results not recorded and 1 incorrect execution of the RADT leaving 2,028 (99.80%) samples from 1,849 individuals eligible for analysis. SARS-CoV-2 was detected in 210 samples by RT-qPCR representing a study prevalence of 10.36% (Fig. 1A). Data on symptoms were obtained and analyzed for 1,676 (82.64%) of 2,028 samples. At the time of sampling 866 (42.70%) swabs were taken from symptomatic individuals, while 810 (39.94%) specimens were collected from asymptomatic individuals. For 352 (17.35%) samples, the symptom status of the respective patients was unknown at the time of analysis. For 599 specimens (69.17%), individuals reported up to three symptoms, for 247 (28.52%) more than three symptoms, and for 20 samples (2.31%) the number of symptoms was not reported by the respective subject. In our cohort 320 (15.78%) samples were obtained from 141 (7.62%) individuals who were tested twice or more (Fig. 1B). A total of 1,239 (61.09%) samples were taken from female and 789 (38.91%) from male individuals. Participants had a median age of 32.25 years (interquartile range [IQR] = 26.15 to 43.12) (Fig. 1C). The median adjusted *C_T_* value determined by RT-qPCR was 31.49 (IQR = 24.19 to 34.16) (Fig. 1D). Of 210 RT-qPCR-positive and 2 RT-qPCR-inconclusive samples, 126 (60%) were cultivated in Vero E6 cells on the same day of sample collection (Fig. 1A). Of these, 8 (4.80%) were excluded due to culture contaminations (*n* = 6) or negative RT-qPCR results upon retesting of inconclusive samples (*n* = 2) (Fig. 1B). A detailed cohort description can be found in Table S1 in the supplemental material.

### Reliable detection of high RNA load samples by RADT.

**FIG 2:**
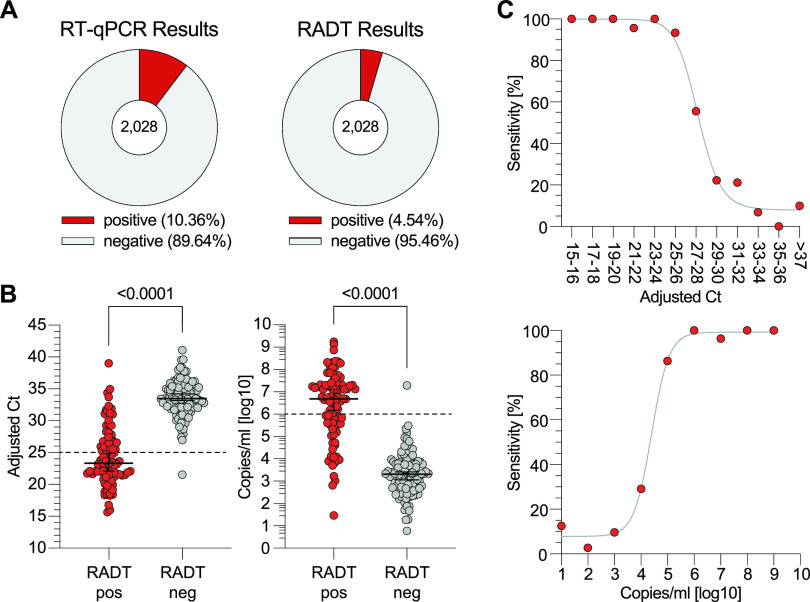
Head-to-head comparison of SARS-CoV-2 detection by RT-qPCR and RADT. (A) RT-qPCR and RADT results of all 2,028 specimens. (B) The 210 RT-qPCR-positive samples are plotted by adjusted *C_T_* values and SARS-CoV-2 RNA load, respectively, and stratified by their RADT result (*P* < 0.0001, MWU). (C) The sensitivity of the RADT is stratified by adjusted *C_T_* values and RNA load in log copies/ml. The top graph shows data points for two *C_T_* units each. Due to low sample sizes, *C_T_* values of >37 were combined.

The Standard Q COVID-19 Ag test yielded a positive result in 92 (4.54%) and a negative result in 1,936 (95.46%) samples (Fig. 2A). Using the results of the RT-qPCR as a reference, RADT classified 90 samples (4.44%) as true positive, 1,816 (89.54%) as true negative, 120 (5.92%) as false negative, and 2 (0.10%) as false positive, resulting in an overall sensitivity and specificity of 42.86% (95% CI = 36.35 to 49.62) and 99.89% (95% CI = 99.60 to 99.98), respectively (Table 1). For positive RADT results, the median *C_T_* was 23.32 (IQR = 21.48 to 26.69) with a median copy number/ml of 6.69 log_10_ (IQR = 5.57 log_10_ to 7.3 log_10_) compared to 33.46 (IQR = 32.04 to 35.38) and 3.3 log_10_ (IQR = 2.66 log_10_ to 3.79 log_10_) for negative RADT results (*P* < 0.0001; Fig. 2B). Stratified by adjusted *C_T_* values the RADT had sensitivities of 100% (14/14), 98.25% (56/57), 88.64% (78/88), and 50.57% (89/176) for adjusted *C_T_* values of <20, <25, <30, and <35, respectively (Table 1). Diagnostic sensitivities of 93.33, 55.55, and 22.22% are reached for adjusted *C_T_* values between 25 and 26, 27 and 28, and 29 and 30. Sensitivities of 86.36, 29.17, and 9.68% are reached for 6 log_10_, 5 log_10_, and 4 log_10_ copies/ml, respectively (Fig. 2C). We conclude that observed RADT sensitivity declines at adjusted *C_T_* values above 27 or below 6 log_10_ copies/ml. However, the RADT reliably detects samples with higher RNA loads.

**TABLE 1:** Performance data of the Standard Q COVID-19 Ag test^*a*^

Subgroup	Total no.
		Sensitivity [% (95% CI)]
Overall^*b*^	2,028	42.86 (36.35–49.62)
*C_T_* < 20 (≙ >7.80 log_10_ copies/ml)	14	100.00 (72.25–100.00)
*C_T_* < 25 (≙ >6.13 log_10_ copies/ml)	57	98.25 (90.71–99.91)
*C_T_* < 30 (≙ >4.46 log_10_ copies/ml)	88	88.64 (80.33–93.71)
*C_T_* < 35 (≙ >2.80 log_10_ copies/ml)	176	50.57 (43.25–57.86)
Symptomatic	866	55.39 (46.81–63.65)
Asymptomatic	810	22.50 (14.73–32.79)

a The RADT sensitivity, specificity, and positive and negative predictive values (PPV and NPV) were calculated using RT-qPCR as a reference. RADT sensitivity was stratified by cycle threshold (*C_T_*) values and symptom status.

b PPV, 97.83% (95% CI = 92.42 to 99.61); NPV, 93.80% (95% CI = 92.64 to 94.79); specificity, 99.89 (95% CI = 99.60 to 99.98).

### Decreased RADT sensitivity over the course of symptom duration.

**FIG 3:**
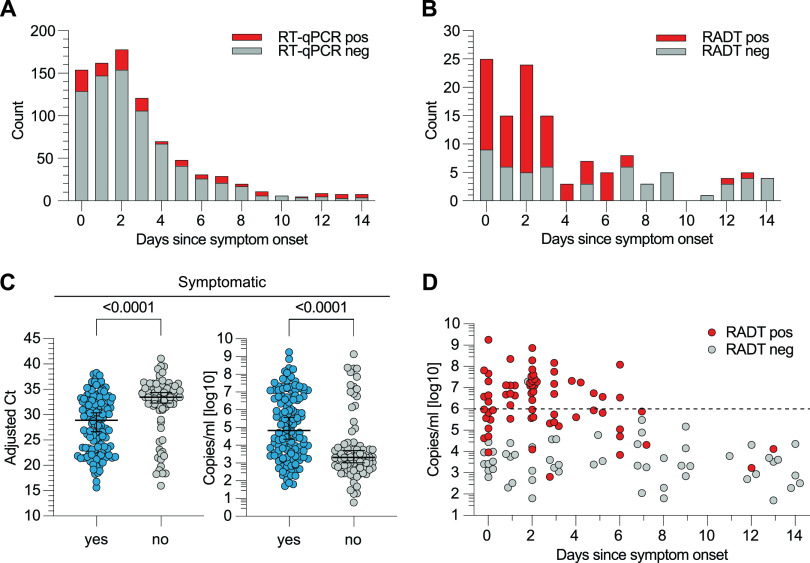
RT-qPCR and RADT results in symptomatic and asymptomatic individuals. (A) The number of RT-qPCR-positive and -negative specimens from symptomatic individuals is plotted by the days since symptom onset. (B) The number of RADT-positive and -negative specimens within the RT-qPCR-positive samples of symptomatic participants (*n* = 130) is stratified by the days since symptom onset. (C) Symptomatic and asymptomatic RT-qPCR positives are plotted by adjusted *C_T_* value and RNA load (*P* < 0.0001, MWU). (D) RT-qPCR-positive samples of symptomatic individuals are plotted by RNA load and stratified by the number of days since symptom onset. The RADT result is indicated in corresponding colors.

We determined that 130 (15.01%) and 73 (8.43%) of 866 samples from symptomatic subjects tested positive in RT-qPCR and RADT, respectively. Symptom duration at the time of sampling was reported for 860 (99.31%) samples with a median duration of 2 days since symptom onset for both RT-qPCR-positive (IQR = 1 to 6) and -negative (IQR = 1 to 4) samples (Fig. 3A). Of samples tested positive by RT-qPCR, RADT detected 56 (68.29%) samples within 4 days since symptom onset. When reported, median symptom duration for RT-qPCR-positive samples with either a RADT-positive (*n* = 69) or -negative (*n* = 55) result was 2 days (IQR = 1 to 3; Fig. 3B). For samples from symptomatic patients, the median adjusted *C_T_* and median copies/ml were 28.90 (IQR = 23.17 to 32.80) and 4.83 log_10_ (IQR = 3.53 log_10_ to 6.74 log_10_), respectively. In individuals not reporting any symptoms, detected RNA load was significantly lower (median adjusted *C_T_* = 33.45 [IQR = 31.26 to 35.31]; median copies/ml = 3.31 log_10_ [IQR = 2.69 log_10_ to 4.05 log_10_; *P* < 0.0001]) (Fig. 3C). Samples with RNA concentrations of >6 log_10_ were only observed up to 6 days after symptom onset. Thus, we conclude that the sensitivity of the RADT decreased with symptom duration as RNA loads declined (Fig. 3D).

### Negative RADT result corresponds with lack of viral cultivability.

**FIG 4:**
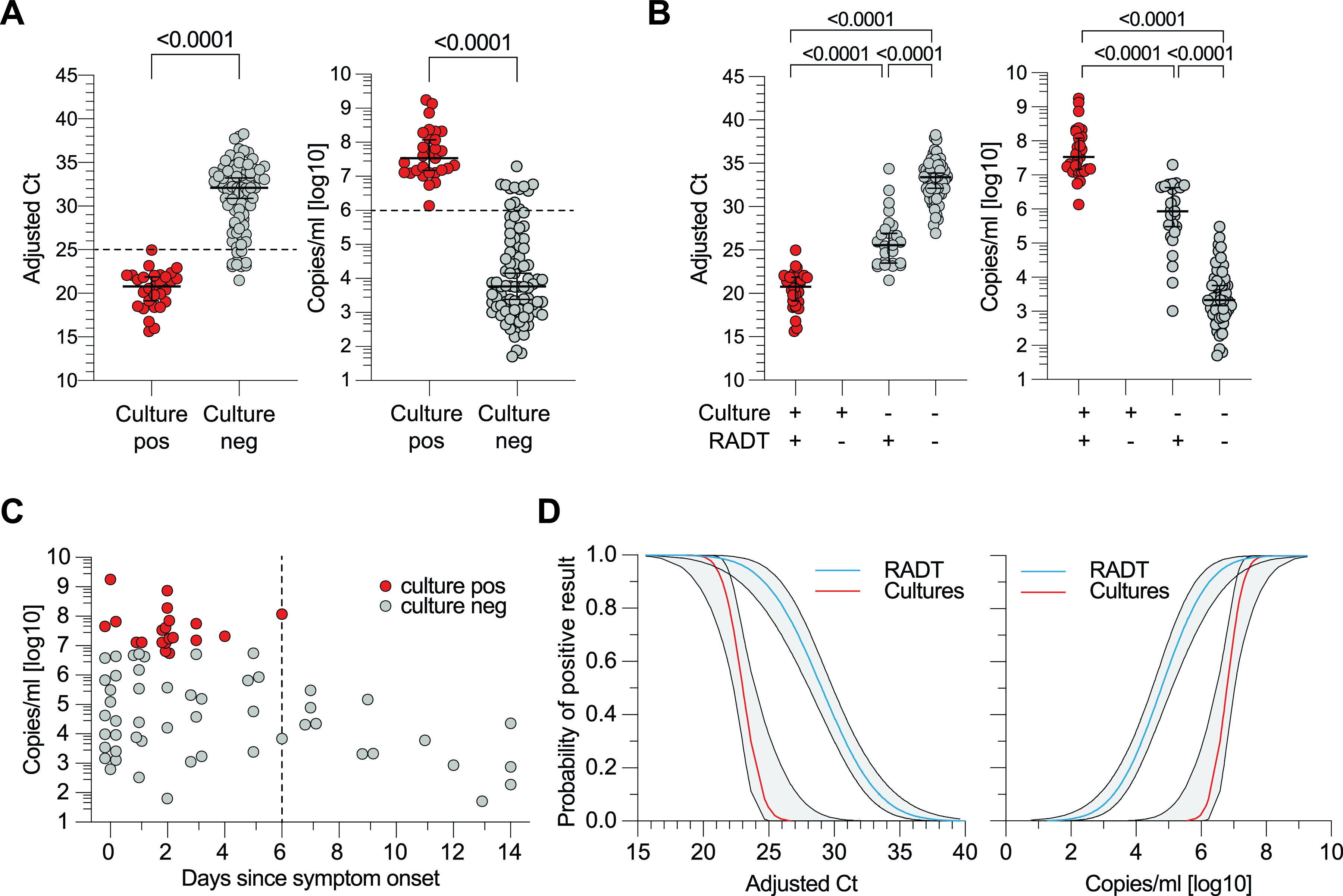
Virus culture analysis and RADT performance in the detection of replication-competent SARS-CoV-2. (A) Positive and negative viral cultures plotted by adjusted *C_T_* and RNA load of the original swab medium (*P* < 0.0001, MWU). (B) Adjusted *C_T_* values and RNA loads of cultured samples are stratified by culture and RADT result (*P* < 0.0001, MWU). (C) Culture-positive and -negative samples are plotted by RNA load of original swab medium and stratified by the days since symptom onset. (D) Probability of positive result for RADT and viral cultures in the context of adjusted *C_T_* values and RNA load (Probit-Model, R-function GLM).

For 118 inoculated cultures, CPE was observed in 29 (24.58%). To confirm virus replication, RT-qPCR of culture supernatant taken on days 4 and 7 was performed as described previously. The observed CPE and positive RT-qPCR results matched in 116 (98.30%) cases. Since CPE is operator dependent, RT-qPCR results were used for further analysis (Table 2). Initial *C_T_* values and copies/ml of positive cultures ranged from 15.64 to 24.97 and 6.14 log_10_ to 9.25 log_10_, whereas negative cultures ranged from 21.5 to 38.27 or 1.71 log_10_ to 7.30 log_10_, respectively (Fig. 4A). All (29/29) positive SARS-CoV-2 cultures had been previously identified as positive by RADT. Of 89 negative cultures, 64 (71.91%) had been previously classified as RADT negative and 25 (28.09%) as RADT positive, resulting in a sensitivity of 100.00% (95% CI = 88.30 to 100.00) and a specificity of 71.91% (95% CI = 61.82 to 80.19) for detecting viral cultivability by RADT (*P* < 0.0001; Table 2). Therefore, the calculated PPV of RADT for viral cultivability in Vero E6 cells was 53.70% (95% CI = 40.61 to 66.31) and the NPV was 100% (95% CI = 94.34 to 100.00; Table 2). For RADT- and culture-positive results, the median adjusted *C_T_* was 20.8 and the median RNA load in copies/ml was 7.53 log_10_ compared to 33.39 and 3.34 log_10_ for RADT- and culture-negative results. The median adjusted *C_T_* and copies/ml for RADT-positive but culture-negative results were 25.43 (IQR = 23.39 to 27.72) and 5.99 log_10_ (IQR = 5.23 log_10_ to 6.67 log_10_), respectively (Fig. 4B). With viral RNA declining over the course of disease, the ability to isolate virus decreased, with no positive culture after 6 days since symptom onset (Fig. 4C). Probit regression of RADT and cell culture analyses show greater probabilities of positive result (PPR) for low adjusted *C_T_* values and high RNA loads, respectively. The virus culture assay shows 90 and 50% PPR for an adjusted *C_T_* value around 21.45 or 7.31 log_10_ copies/ml and 23 or 6.8 log_10_ copies/ml, respectively. RADT show a PPR of 90 and 50% at an adjusted *C_T_* value of 24.7 or 6.24 log_10_ copies/ml and 29.0 or 4.78 log_10_ copies/ml, respectively (Fig. 4D). In summary, these data show that a negative RADT result can reliably predict noninfectiousness in Vero E6 cells.

**TABLE 2:** Comparison of RADT and culture results^*a*^

Parameter	Culture positive	Culture negative	Total
No.			
RADT positive	29	25	54
RADT negative	0	64	64
Total	29	89	118
			
%			
Sensitivity	100.00 (88.30–100.00)	PPV	53.70 (40.61–66.31)
Specificity	71.91 (61.82–80.20)	NPV	100.00 (94.34–100.00)

a Analysis of RADT performance in the context of culture infectivity (*P* < 0.0001, Fisher exact test; weighted analysis *P* < 0.0001; 95% CI values are indicated in parentheses).

### Adjustments for repeated testing reveal no statistically significant difference.

To evaluate whether repeated testing of several individuals affects our conclusions, additional statistical analyses were carried out (see Materials and Methods). Correction for repeated RADT measurements was based on weighted counts (see Fig. S2A) rounded to whole numbers, yielding very similar results compared to the unweighted variant (see Fig. S2). In addition, the performance of the RADT was analyzed for the first measurement of each subject only (simplified analysis; Fig. S2A). Calculated sensitivities showed almost identical values as overall sensitivities described above (see Fig. S2B to D). For all studies that applied an MWU test (Fig. 2B, 3C, and 4A and B), the *P* values remain *P* < 0.0001 according a generalized estimating equation (GEE).

## DISCUSSION

RADT are inexpensive and fast diagnostic tools that can be immediately performed at the point of care. Here, we present comprehensive data on the use of Standard Q COVID-19 RADT for high-throughput testing of a large cohort tested under real-life conditions in a SARS-CoV-2 outpatient diagnostic center.

Implementation and execution of the RADT testing was completed without any difficulties. Upon following the manufacturer’s instructions, there were only two cases in which the result could not be read out clearly. As a consequence, we were able to conduct 2,028 paired RT-qPCR and RADT tests directly on site and cultivate virus on the same day without prior sample freezing. While the sensitivities of other RADT vary between 24 and 93% in different studies (33–35), the reported Standard Q RADT sensitivities are mostly in the range from 68 to 90% (14–24). The overall diagnostic sensitivity observed in our study was 42.86%. However, the investigated cohort of nonhospitalized patients was to a large extent comprised of individuals with adjusted *C_T_* values over 30 (122/210). Stratification by RNA load revealed that the Standard Q RADT performed reliably for patients with RNA loads over 5.4 log_10_ or below *C_T_* 27, which is in accordance to recent studies (14, 23, 25, 36). Since an RNA concentration of 6 log_10_ copies/ml is commonly suspected to be the threshold for contagiousness, we aimed to investigate the correlation between RADT result and SARS-CoV-2 *in vitro* infectivity (28, 29).

In contrast to highly sensitive nucleic acid-based detection methods that do not specifically test for intact viral particles required for transmission, viral culture is a commonly used, albeit laborious, method outside of routine diagnostics to determine the presence of infectious virus in samples (7, 30–32, 37). Although the detection of viable virus in cell culture models is strong evidence of infectiousness, a negative result does not eliminate the possibility of human transmission (11, 38). Moreover, the validity of viral culture as a surrogate for infectivity may depend on the susceptibility of the cell line used (31, 32, 37). However, loss of infectious titer in classical Vero E6 cells has been associated with a lack of transmission despite detection of viral RNA in preclinical models (39).

To investigate whether the Standard Q RADT might be able to reliably detect culture-positive samples in Vero E6 cells, we attempted virus isolation from samples positive for SARS-CoV-2 RNA. All samples from which virus could be recovered had previously tested positive in RADT. Moreover, none of the samples tested negative by RADT contained infectious virus determined by cell culture. Furthermore, when taking symptom duration into account, we detected no positive culture 6 days after symptom onset, indicating a decreased probability of recovering viable virus as symptom duration increases (29, 30, 40–42); at the same time, the RADT identified positive samples for up to 9 days. While some groups have described virus isolation from samples above *C_T_* 30 (31, 43), our study only observed positive cultures from samples with higher RNA loads, which is in accordance with previous observations (10, 36, 41, 44). Taking the time of suspected exposure and duration of symptoms into account (31, 38), our results suggest that RADT testing is of potential use for estimating infectivity at the time of sampling.

This study, however, is subject to some limitations. Although the examined single-center study population was large, our cohort might not be considered representative of the general population due to young age and disproportionate gender distribution. The data on symptoms and their duration are only reliable to a limited extent, since they were retrospectively analyzed from mostly self-reported symptoms entered into a web tool. Furthermore, instead of a nasal swab, we used a combined oro- and nasopharyngeal swab to investigate RADT performance, which impedes the feasibility for the general public. Lastly, although we have corrected for using different SARS-CoV-2 detection assays by reporting data in RNA loads, the diverse set of amplification methods remains a limitation.

In combating overdispersed SARS-CoV-2 transmission, rapid detection and isolation of highly infectious individuals is a primary goal (8, 9, 45–49). In our investigation the Standard Q RADT was able to reliably detect high RNA loads, as well as all culture-positive samples. Therefore, this test could be used as a fast surrogate marker for viral cultivation to identify and prevent SARS-CoV-2 transmissions by highly infectious individuals. Although less sensitive than RT-qPCR and therefore less suited for an inpatient setting, RADT could compensate for this disadvantage through easy and feasible mass screenings in a community setting (8, 50). Furthermore, one might suspect that RT-qPCR positive, but RADT-negative individuals do not pose a high risk of transmissions, since all samples remained culture negative in our experimental setup. However, individual results must be interpreted with caution, as SARS-CoV-2 infection could remain undetected in early stages. In summary, our results suggest that SARS-CoV-2 transmission could be reduced by systematic RADT, despite the fact that some infected individuals will not be detected by the test (51). Simple to perform and applicable anywhere, RADT enable mass testing as a complementary method to RT-qPCR to more effectively combat the SARS-CoV-2 pandemic.

## References

[1] Fraser C, Riley S, Anderson RM, Ferguson NM. 2004. Factors that make an infectious disease outbreak controllable. Proc Natl Acad Sci U S A 101:6146–6151. 10.1073/pnas.0307506101. 15071187 PMC395937

[2] Hellewell J, Abbott S, Gimma A, Bosse NI, Jarvis CI, Russell TW, Munday JD, Kucharski AJ, Edmunds WJ, Funk S, Eggo RM, Sun F, Flasche S, Quilty BJ, Davies N, Liu Y, Clifford S, Klepac P, Jit M, Diamond C, Gibbs H, van Zandvoort K. 2020. Feasibility of controlling COVID-19 outbreaks by isolation of cases and contacts. Lancet Glob Health 8:e488–e496. 10.1016/S2214-109X(20)30074-7. 32119825 PMC7097845

[3] Kretzschmar ME, Rozhnova G, Bootsma MCJ, van Boven M, van de Wijgert JHHM, Bonten MJM. 2020. Impact of delays on effectiveness of contact tracing strategies for COVID-19: a modeling study. Lancet Public Health 5:e452–e459. 10.1016/S2468-2667(20)30157-2. 32682487 PMC7365652

[4] Corman VM, Landt O, Kaiser M, Molenkamp R, Meijer A, Chu DK, Bleicker T, Brünink S, Schneider J, Schmidt ML, Mulders DG, Haagmans BL, van der Veer B, van den Brink S, Wijsman L, Goderski G, Romette J-L, Ellis J, Zambon M, Peiris M, Goossens H, Reusken C, Koopmans MP, Drosten C. 2020. Detection of 2019 novel coronavirus (2019-nCoV) by real-time RT-PCR. Eurosurveillance 25:20000045. 10.2807/1560-7917.ES.2020.25.3.2000045. PMC6988269 31992387

[5] Sun J, Xiao J, Sun R, Tang X, Liang C, Lin H, Zeng L, Hu J, Yuan R, Zhou P, Peng J, Xiong Q, Cui F, Liu Z, Lu J, Tian J, Ma W, Ke C. 2020. Prolonged persistence of SARS-CoV-2 RNA in body fluids. Emerg Infect Dis 26:1834–1838. 10.3201/eid2608.201097. 32383638 PMC7392422

[6] Cevik M, Kuppalli K, Kindrachuk J, Peiris M. 2020. Virology, transmission, and pathogenesis of SARS-CoV-2. BMJ 371:m3862. 10.1136/bmj.m3862. 33097561

[7] Huang C-G, Lee K-M, Hsiao M-J, Yang S-L, Huang P-N, Gong Y-N, Hsieh T-H, Huang P-W, Lin Y-J, Liu Y-C, Tsao K-C, Shih S-R. 2020. Culture-based virus isolation to evaluate potential infectivity of clinical specimens tested for COVID-19. J Clin Microbiol 58:e01068-20. 10.1128/JCM.01068-20. 32518072 PMC7383522

[8] Mina MJ, Parker R, Larremore DB. 2020. Rethinking Covid-19 test sensitivity: a strategy for containment. N Engl J Med 383:e120. 10.1056/NEJMp2025631. 32997903

[9] Mina MJ, Peto TE, García-Fiñana M, Semple MG, Buchan IE. 2021. Clarifying the evidence on SARS-CoV-2 antigen rapid tests in public health responses to COVID-19. Lancet 397:1425–1427. 10.1016/S0140-6736(21)00425-6. 33609444 PMC8049601

[10] Pray IW, Ford L, Cole D, Lee C, Bigouette JP, Abedi GR, Bushman D, Delahoy MJ, Currie D, Cherney B, Kirby M, Fajardo G, Caudill M, Langolf K, Kahrs J, Kelly P, Pitts C, Lim A, Aulik N, Tamin A, Harcourt JL, Queen K, Zhang J, Whitaker B, Browne H, Medrzycki M, Shewmaker P, Folster J, Bankamp B, Bowen MD, Thornburg NJ, Goffard K, Limbago B, Bateman A, Tate JE, Gieryn D, Kirking HL, Westergaard R, Killerby M, Jiang B, Vinjé J, Hopkins AL, Katz E, Barclay L, Esona M, Gautam R, Mijatovic-Rustempasic S, Moon S-S, Bessey T, Chhabra P, CDC COVID-19 Surge Laboratory Group, et al. 2021. Performance of an antigen-based test for asymptomatic and symptomatic SARS-CoV-2 testing at two university campuses: Wisconsin, September–October 2020. MMWR Morb Mortal Wkly Rep 69:1642–1647. 10.15585/mmwr.mm695152a3. 33382679 PMC9191905

[11] Prince-Guerra JL, Almendares O, Nolen LD, Gunn JKL, Dale AP, Buono SA, Deutsch-Feldman M, Suppiah S, Hao L, Zeng Y, Stevens VA, Knipe K, Pompey J, Atherstone C, Bui DP, Powell T, Tamin A, Harcourt JL, Shewmaker PL, Medrzycki M, Wong P, Jain S, Tejada-Strop A, Rogers S, Emery B, Wang H, Petway M, Bohannon C, Folster JM, MacNeil A, Salerno R, Kuhnert-Tallman W, Tate JE, Thornburg NJ, Kirking HL, Sheiban K, Kudrna J, Cullen T, Komatsu KK, Villanueva JM, Rose DA, Neatherlin JC, Anderson M, Rota PA, Honein MA, Bower WA. 2021. Evaluation of Abbott BinaxNOW rapid antigen test for SARS-CoV-2 infection at two community-based testing sites: Pima County, Arizona, November 3–17, 2020. MMWR Morb Mortal Wkly Rep 70:100–105. 10.15585/mmwr.mm7003e3. 33476316 PMC7821766

[12] Lee J, Kim SY, Huh HJ, Kim N, Sung H, Lee H, Roh KH, Kim TS, Hong KH. 2021. Clinical performance of the standard Q COVID-19 rapid antigen test and simulation of its real-world application in Korea. Ann Lab Med 41:588–592. 10.3343/alm.2021.41.6.588. 34108286 PMC8203442

[13] Jääskeläinen AE, Ahava MJ, Jokela P, Szirovicza L, Pohjala S, Vapalahti O, Lappalainen M, Hepojoki J, Kurkela S. 2021. Evaluation of three rapid lateral flow antigen detection tests for the diagnosis of SARS-CoV-2 infection. J Clin Virol 137:104785. 10.1016/j.jcv.2021.104785. 33711694 PMC7934791

[14] Kohmer N, Toptan T, Pallas C, Karaca O, Pfeiffer A, Westhaus S, Widera M, Berger A, Hoehl S, Kammel M, Ciesek S, Rabenau HF. 2021. The comparative clinical performance of four SARS-CoV-2 rapid antigen tests and their correlation to infectivity *in vitro*. J Clin Microbiol 10:328. 10.3390/jcm10020328. PMC7830733 33477365

[15] Nalumansi A, Lutalo T, Kayiwa J, Watera C, Balinandi S, Kiconco J, Nakaseegu J, Olara D, Odwilo E, Serwanga J, Kikaire B, Ssemwanga D, Nabadda S, Ssewanyana I, Atwine D, Mwebesa H, Bosa HK, Nsereko C, Cotten M, Downing R, Lutwama J, Kaleebu P. 2021. Field evaluation of the performance of a SARS-CoV-2 antigen rapid diagnostic test in Uganda using nasopharyngeal samples. Int J Infect Dis 104:282–286. 10.1016/j.ijid.2020.10.073. 33130198 PMC7836828

[16] Iglὁi Z, Velzing J, van Beek J, van de Vijver D, Aron G, Ensing R, Benschop K, Han W, Boelsums T, Koopmans M, Geurtsvankessel C, Molenkamp R. 2021. Clinical evaluation of Roche SD Biosensor Rapid Antigen Test for SARS-CoV-2 in municipal health service testing site, the Netherlands. Emerg Infect Dis 27:1323–1329. 10.3201/eid2705.204688. 33724916 PMC8084500

[17] Corman VM, Haage VC, Bleicker T, Schmidt ML, Mühlemann B, Zuchowski M, Jo WK, Tscheak P, Möncke-Buchner E, Müller MA, Krumbholz A, Drexler JF, Drosten C. 2021. Comparison of seven commercial SARS-CoV-2 rapid point-of-care antigen tests: a single-centre laboratory evaluation study. Lancet Microbe 2:e311–e319. 10.1016/S2666-5247(21)00056-2. 33846704 PMC8026170

[18] Möckel M, Corman VM, Stegemann MS, Hofmann J, Stein A, Jones TC, Gastmeier P, Seybold J, Offermann R, Bachmann U, Lindner T, Bauer W, Drosten C, Rosen A, Somasundaram R. 2021. SARS-CoV-2 antigen rapid immunoassay for diagnosis of COVID-19 in the emergency department. Biomarkers 26:213–220. 10.1080/1354750X.2021.1876769. 33455451 PMC7898296

[19] Ristić M, Nikolić N, Čabarkapa V, Turkulov V, Petrović V. 2021. Validation of the STANDARD Q COVID-19 antigen test in Vojvodina, Serbia. PLoS One 16:e0247606. 10.1371/journal.pone.0247606. 33617597 PMC7899368

[20] Cerutti F, Burdino E, Milia MG, Allice T, Gregori G, Bruzzone B, Ghisetti V. 2020. Urgent need of rapid tests for SARS CoV-2 antigen detection: evaluation of the SD-Biosensor antigen test for SARS-CoV-2. J Clin Virol 132:104654. 10.1016/j.jcv.2020.104654. 33053494 PMC7522649

[21] Turcato G, Zaboli A, Pfeifer N, Ciccariello L, Sibilio S, Tezza G, Ausserhofer D. 2021. Clinical application of a rapid antigen test for the detection of SARS-CoV-2 infection in symptomatic and asymptomatic patients evaluated in the emergency department: a preliminary report. J Infect 82:e14–e16. 10.1016/j.jinf.2020.12.012. PMC7748975 33347944

[22] Caruana G, Croxatto A, Kampouri E, Kritikos A, Opota O, Foerster M, Brouillet R, Senn L, Lienhard R, Egli A, Pantaleo G, Carron P-N, Greub G. 2021. Implementing SARS-CoV-2 rapid antigen testing in the emergency ward of a Swiss university hospital: the INCREASE Study. Microorganisms 9:798. 10.3390/microorganisms9040798. 33920307 PMC8069749

[23] Berger A, Nsoga MTN, Perez-Rodriguez FJ, Aad YA, Sattonnet-Roche P, Gayet-Ageron A, Jaksic C, Torriani G, Boehm E, Kronig I, Sacks JA, de Vos M, Bausch FJ, Chappuis F, Renzoni A, Kaiser L, Schibler M, Eckerle I. 2021. Diagnostic accuracy of two commercial SARS-CoV-2 antigen-detecting rapid tests at the point of care in community-based testing centers. PLoS One 16:e0248921. 10.1371/journal.pone.0248921. 33788882 PMC8011749

[24] Peña‐Rodríguez M, Viera‐Segura O, García‐Chagollán M, Zepeda‐Nuño JS, Muñoz‐Valle JF, Mora‐Mora J, Espinoza‐De León G, Bustillo‐Armendáriz G, García‐Cedillo F, Vega‐Magaña N. 2021. Performance evaluation of a lateral flow assay for nasopharyngeal antigen detection for SARS‐CoV‐2 diagnosis. J Clin Lab Anal 35:e23745. 10.1002/jcla.23745. 33675086 PMC8128319

[25] Pilarowski G, Marquez C, Rubio L, Peng J, Martinez J, Black D, Chamie G, Jones D, Jacobo J, Tulier-Laiwa V, Rojas S, Rojas S, Cox C, Nakamura R, Petersen M, DeRisi J, Havlir DV. 2020. Field performance and public health response using the BinaxNOW TM Rapid SARS-CoV-2 antigen detection assay during community-based testing. Clin Infect Dis 2020:ciaa1890. 10.1093/cid/ciaa1890. PMC7799223 33367619

[26] Dinnes J, Deeks JJ, Berhane S, Taylor M, Adriano A, Davenport C, Dittrich S, Emperador D, Takwoingi Y, Cunningham J, Beese S, Domen J, Dretzke J, Ferrante di Ruffano L, Harris IM, Price MJ, Taylor-Phillips S, Hooft L, Leeflang MM, McInnes MD, Spijker R, Van den Bruel A, Cochrane COVID-19 Diagnostic Test Accuracy Group. 2021. Rapid, point-of-care antigen and molecular-based tests for diagnosis of SARS-CoV-2 infection. Cochrane Database of Systematic Rev 3:CD013705. 10.1002/14651858.CD013705.pub2. PMC8078597 33760236

[27] Marks M, Millat-Martinez P, Ouchi D, Roberts C, Alemany A, Corbacho-Monné M, Ubals M, Tobias A, Tebé C, Ballana E, Bassat Q, Baro B, Vall-Mayans M, G-Beiras C, Prat N, Ara J, Clotet B, Mitjà O. 2021. Transmission of COVID-19 in 282 clusters in Catalonia, Spain: a cohort study. Lancet Infect Dis 21:629–636. 10.1016/S1473-3099(20)30985-3. 33545090 PMC7906723

[28] van Kampen JJA, van de Vijver DAMC, Fraaij PLA, Haagmans BL, Lamers MM, Okba N, van den Akker JPC, Endeman H, Gommers D, Cornelissen JJ, Hoek RAS, van der Eerden MM, Hesselink DA, Metselaar HJ, Verbon A, de Steenwinkel JEM, Aron GI, van Gorp ECM, van Boheemen S, Voermans JC, Boucher CAB, Molenkamp R, Koopmans MPG, Geurtsvankessel C, van der Eijk AA. 2021. Duration and key determinants of infectious virus shedding in hospitalized patients with coronavirus disease-2019 (COVID-19). Nat Commun 12:267. 10.1038/s41467-020-20568-4. 33431879 PMC7801729

[29] Wölfel R, Corman VM, Guggemos W, Seilmaier M, Zange S, Müller MA, Niemeyer D, Jones TC, Vollmar P, Rothe C, Hoelscher M, Bleicker T, Brünink S, Schneider J, Ehmann R, Zwirglmaier K, Drosten C, Wendtner C. 2020. Virological assessment of hospitalized patients with COVID-2019. Nature 581:465–469. 10.1038/s41586-020-2196-x. 32235945

[30] ECDC. 2020. Guidance for discharge and ending of isolation of people with COVID-19. Technical report. European Centre for Disease Prevention and Control, Stockholm, Sweden.

[31] Jefferson T, Spencer EA, Brassey J, Heneghan C. 2020. Viral cultures for COVID-19 infectious potential assessment: a systematic review. Clin Infect Dis 2020:ciaa1764. 10.1093/cid/ciaa1764. PMC7799320 33270107

[32] Basile K, McPhie K, Carter I, Alderson S, Rahman H, Donovan L, Kumar S, Tran T, Ko D, Sivaruban T, Ngo C, Toi C, O’Sullivan MV, Sintchenko V, Chen SC-A, Maddocks S, Dwyer DE, Kok J. 2020. Cell-based culture of SARS-CoV-2 informs infectivity and safe de-isolation assessments during COVID-19. Clin Infect Dis 2020:ciaa1579. 10.1093/cid/ciaa1579. PMC7665383 33098412

[33] Diao B, Wen K, Zhang J, Chen J, Han C, Chen Y, Wang S, Deng G, Zhou H, Wu Y. 2021. Accuracy of a nucleocapsid protein antigen rapid test in the diagnosis of SARS-CoV-2 infection. Clin Microbiol Infect 27:289.e1–289.e4. 10.1016/j.cmi.2020.09.057. PMC7534827 33031947

[34] Linares M, Pérez-Tanoira R, Carrero A, Romanyk J, Pérez-García F, Gómez-Herruz P, Arroyo T, Cuadros J. 2020. Panbio antigen rapid test is reliable to diagnose SARS-CoV-2 infection in the first 7 days after the onset of symptoms. J Clin Virol 133:104659. 10.1016/j.jcv.2020.104659. 33160179 PMC7561603

[35] Lambert-Niclot S, Cuffel A, Le Pape S, Vauloup-Fellous C, Morand-Joubert L, Roque-Afonso A-M, Le Goff J, Delaugerre C. 2020. Evaluation of a rapid diagnostic assay for detection of SARS-CoV-2 antigen in nasopharyngeal swabs. J Clin Microbiol 58:e00977-20. 10.1128/JCM.00977-20. 32404480 PMC7383555

[36] Strömer A, Rose R, Schäfer M, Schön F, Vollersen A, Lorentz T, Fickenscher H, Krumbholz A. 2020. Performance of a point-of-care test for the rapid detection of SARS-CoV-2 antigen. Microorganisms 9:58. 10.3390/microorganisms9010058. PMC7823488 33379279

[37] Pekosz A, Parvu V, Li M, Andrews JC, Manabe YC, Kodsi S, Gary DS, Roger-Dalbert C, Leitch J, Cooper CK. 2021. Antigen-based testing but not real-time polymerase chain reaction correlates with severe acute respiratory syndrome coronavirus 2 viral culture. Clin Infect Dis 2021:ciaa1706. 10.1093/cid/ciaa1706. PMC7929138 33479756

[38] Rhee C, Kanjilal S, Baker M, Klompas M. 2021. Duration of severe acute respiratory syndrome coronavirus 2 (SARS-CoV-2) infectivity: when is it safe to discontinue isolation? Clin Infect Dis 72:1467–1474. 10.1093/cid/ciaa1249. 33029620 PMC7499497

[39] Sia SF, Yan L-M, Chin AWH, Fung K, Choy K-T, Wong AYL, Kaewpreedee P, Perera RAPM, Poon LLM, Nicholls JM, Peiris M, Yen H-L. 2020. Pathogenesis and transmission of SARS-CoV-2 in golden hamsters. Nature 583:834–838. 10.1038/s41586-020-2342-5. 32408338 PMC7394720

[40] Cevik M, Tate M, Lloyd O, Maraolo AE, Schafers J, Ho A. 2021. SARS-CoV-2, SARS-CoV, and MERS-CoV viral load dynamics, duration of viral shedding, and infectiousness: a systematic review and meta-analysis. Lancet Microbe 2:e13–e22. 10.1016/S2666-5247(20)30172-5. 33521734 PMC7837230

[41] Bullard J, Dust K, Funk D, Strong JE, Alexander D, Garnett L, Boodman C, Bello A, Hedley A, Schiffman Z, Doan K, Bastien N, Li Y, Van Caeseele PG, Poliquin G. 2020. Predicting infectious SARS-CoV-2 from diagnostic samples. Clin Infect Dis 71:2663–2666. 10.1093/cid/ciaa638. 32442256 PMC7314198

[42] Perera RAPM, Tso E, Tsang OTY, Tsang DNC, Fung K, Leung YWY, Chin AWH, Chu DKW, Cheng SMS, Poon LLM, Chuang VWM, Peiris M. 2020. SARS-CoV-2 virus culture and subgenomic RNA for respiratory specimens from patients with mild coronavirus disease. Emerg Infect Dis 26:2701–2704. 10.3201/eid2611.203219. 32749957 PMC7588524

[43] Gniazdowski V, Morris CP, Wohl S, Mehoke T, Ramakrishnan S, Thielen P, Powell H, Smith B, Armstrong DT, Herrera M, Reifsnyder C, Sevdali M, Carroll KC, Pekosz A, Mostafa HH. 2020. Repeat COVID-19 molecular testing: correlation of SARS-CoV-2 culture with molecular assays and cycle thresholds. Clin Infect Dis 2020:ciaa1616. 10.1093/cid/ciaa1616. PMC7665437 33104776

[44] Kim M-C, Cui C, Shin K-R, Bae J-Y, Kweon O-J, Lee M-K, Choi S-H, Jung S-Y, Park M-S, Chung J-W. 2021. Duration of culturable SARS-CoV-2 in hospitalized patients with Covid-19. N Engl J Med 384:671–673. 10.1056/NEJMc2027040. 33503337 PMC7934323

[45] Larremore DB, Wilder B, Lester E, Shehata S, Burke JM, Hay JA, Tambe M, Mina MJ, Parker R. 2021. Test sensitivity is secondary to frequency and turnaround time for COVID-19 screening. Sci Adv 7:eabd5393. 10.1126/sciadv.abd5393. 33219112 PMC7775777

[46] Muller N, Kunze M, Steitz F, Saad NJ, Mühlemann B, Beheim-Schwarzbach JI, Schneider J, Drosten C, Murajda L, Kochs S, Ruscher C, Walter J, Zeitlmann N, Corman VM. 2020. Severe acute respiratory syndrome coronavirus 2 outbreak related to a nightclub, Germany, 2020. Emerg Infect Dis 27:645–648. 10.3201/eid2702.204443. 33263514 PMC7853558

[47] Correa-Martínez CL, Kampmeier S, Kümpers P, Schwierzeck V, Hennies M, Hafezi W, Kühn J, Pavenstädt H, Ludwig S, Mellmann A. 2020. A pandemic in times of global tourism: superspreading and exportation of COVID-19 cases from a ski area in Austria. J Clin Microbiol 58:e00588-20. 10.1128/JCM.00588-20. 32245833 PMC7269389

[48] Adam DC, Wu P, Wong JY, Lau EHY, Tsang TK, Cauchemez S, Leung GM, Cowling BJ. 2020. Clustering and superspreading potential of SARS-CoV-2 infections in Hong Kong. Nat Med 26:1714–1719. 10.1038/s41591-020-1092-0. 32943787

[49] Cevik M, Marcus JL, Buckee C, Smith TC. 2020. Severe acute respiratory syndrome coronavirus 2 (SARS-CoV-2) transmission dynamics should inform policy. Clin Infect Dis 2020:ciaa1442. 10.1093/cid/ciaa1442. PMC7543342 32964919

[50] Lloyd-Smith JO, Schreiber SJ, Kopp PE, Getz WM. 2005. Superspreading and the effect of individual variation on disease emergence. Nature 438:355–359. 10.1038/nature04153. 16292310 PMC7094981

[51] Pavelka M, Van-Zandvoort K, Abbott S, Sherratt K, Majdan M, Analýz IZ, Jarčuška P, Krajčí M, Flasche S, Funk S, CMMID COVID-19 Working Group 5. 2021. The impact of population-wide rapid antigen testing on SARS-CoV-2 prevalence in Slovakia. Science 372:635–641. 10.1126/science.abf9648. 33758017 PMC8139426

