# Supplementary material for: Evaluation of a Rapid Antigen Test To Detect SARS-CoV-2 Infection and Identify Potentially Infectious Individuals
Source: J Clin Microbiol. 2021 Aug 18;59(9):e00896-21. doi: 10.1128/JCM.00896-21 (PMC8373008; doi:10.1128/JCM.00896-21)
Supplement: Supplemental file 1 — Tables S1 and S2 and Fig. S1 and S2. Download JCM.00896-21-s0001.pdf, PDF file, 0.7 MB [file jcm.00896-21-s0001.pdf]

		**Total**	**PCR$^+$**	**PCR$^-$**	**p-value**
N		2,028	210<br>(10.36%)	1,818<br>(89.64%)	-
Median Age (IQR)		32.25<br>(26.15-43.12)	31.55<br>(24.40 - 46.47)	32.36<br>(26.31 - 42.86)	-
Sex	m	789<br>(38.91%)	89<br>(42.38%)	700<br>(38.50%)	0.2954<br>(0.1166)
	w	1,239<br>(61.09%)	121<br>(57.62%)	1,118<br>(61.50%)	
N		1,676	210	1,466	-
Symptomatic	yes	866<br>(51.67%)	130<br>(61.90%)	736<br>(50.20%)	0.0015<br>(0.0013)
	no	810<br>(48.33%)	80<br>(38.10%)	730<br>(49.80%)	
		**Total**	**RADT$^+$**	**RADT$^-$**	**p-value**
N		2,028	92<br>(4.54%)	1936<br>(95.46%)	-
Median Age (IQR)		32.25<br>(26.15-43.12)	31.71<br>(24.33-49.79)	32.28<br>(26.17-42.87)	-
Sex	m	789<br>(38.90%)	43<br>(46.74%)	746<br>(38.53%)	0.1257<br>(0.0945)
	w	1,239<br>(61.10%)	49<br>(53.26%)	1190<br>(61.47%)	
N		1676	92	1584	-
Symptomatic	yes	866<br>(51.67%)	73<br>(79.35%)	793<br>(50.06%)	<0.0001<br>(<0.0001)
	no	810<br>(48.33%)	19<br>(20.65%)	791<br>(49.94%)	
		**Total**	**culture$^+$**	**culture$^-$**	**p-value**
N		118	29<br>(24.58%)	89<br>(75.42%)	-
Median Age (IQR)		32.12<br>(25.61-46.47)	40.65<br>(30.01-52.15)	31.38<br>(25.52-44.84)	-
Sex	m	46<br>(38.98%)	15<br>(51.72%)	31<br>(34.83%)	0.1270<br>(0.1133)
	w	72<br>(61.02%)	14<br>(48.28%)	58<br>(65.17%)	
N		118	29	89	-
Symptomatic	yes	72<br>(61.02%)	20<br>(68.97%)	52<br>(58.43%)	0.3833<br>(0.8183)
	no	46<br>(38.98%)	9<br>(31.03%)	37<br>(41.57%)	

Table S1. Additional cohort description. Test results by age, sex and symptoms (weighted analyses for repeated measures in brackets; all p-values by Fisher's exact test).

		Total	RADT$^+$	RADT$^-$	p-value
N		1676	92	1584	-
Symptomatic	yes	866	73	793	<0.0001
	no	810	19	791	(<0.0001)
N		846	63	783	-
Cough	yes	347	37	310	
		(41.02%)	(58.73%)	(39.59%)	0.0034
	no	499	26	473	(0.0059)
		(58.98%)	(41.27%)	(60.41%)	
Fever	yes	101	24	77	
		(11.94%)	(38.10%)	(9.83%)	<0.0001
	no	745	39	706	(<0.0001)
		(88.06)	(61.90%)	(90.17%)	
Rhinitis	yes	466	41	425	
		(55.08%)	(65.08%)	(54.28%)	0.1141
	no	380	22	358	(0.1806)
		(44.92%)	(34.92%)	(45.72%)	
Loss of taste/smell	yes	83	21	62	
		(9.81%)	(33.33%)	(7.92%)	<0.0001
	no	763	42	721	(<0.0001)
		(90.19%)	(66.67%)	(92.08%)	
Headache	yes	503	49	454	
		(59.46%)	(77.78%)	(57.98%)	0.0020
	no	343	14	329	(0.0024)
		(40.54%)	(22.22%)	(42.02%)	
Sore throat	yes	512	35	477	
		(60.52%)	(55.56%)	(60.92%)	0.4232
	no	334	28	306	(0.3407)
		(39.48%)	(44.44%)	(39.08%)	
Limb pain	yes	205	28	177	
		(24.23%)	(44.44%)	(22.61%)	0.0003
	no	641	35	606	(0.0003)
		(75.77%)	(55.56%)	(77.39%)	
Respiratory problems	yes	48	4	44	
		(5.67%)	(6.35%)	(5.62%)	0.7757
	no	798	59	739	(0.7736)
		(94.33%)	(93.65%)	(94.38%)	
Diarrhea	yes	97	9	88	
		(11.47%)	(14.29%)	(11.24%)	0.4167
	no	749	54	695	(0.6733)
		(88.53%)	(85.71%)	(88.76%)	
N		860	70	790	-
Days since symptom onset	0-2 d	494	45	449	
		(57.44%)	(64.29%)	(56.84%)	
	3-7 d	299	23	276	0.2213
		(34.77%)	(32.86%)	(34.94%)	(0.2597)
	8-14 d	67	2	65	
		(7.79%)	(2.86%)	(8.23%)	
prior test result	yes	666	17	649	
(within 8 weeks before study)		(32.84%)	(18.48%)	(33.52%)	0.0021
	unknown	1362	75	1287	(0.0060)
		(67.16%)	(81.52%)	(66.48%)	

Table S2. RADT results by symptoms and follow-up testing (weighted analyses for repeated measures in brackets; all p-values by Fisher's exact test).

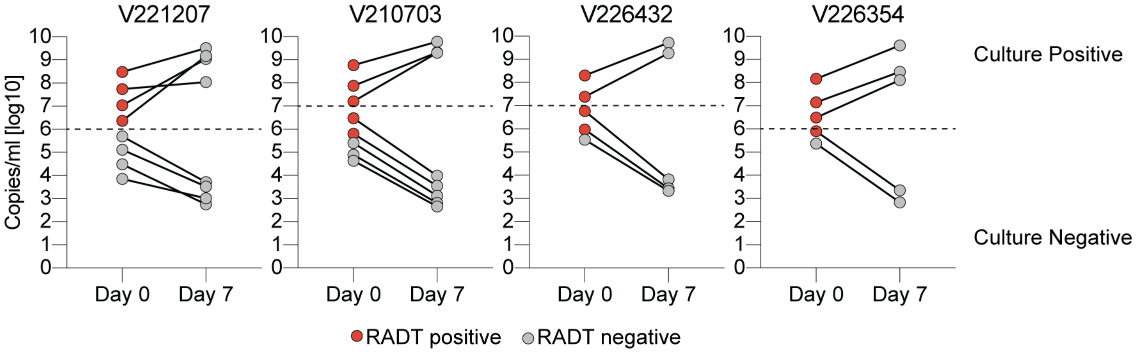

Fig. S1. Virus cultivation of 1:5 dilutions.

Samples of four different individuals with high viral loads were diluted and subsequently used for RADT testing and virus cultivation. Culture supernatant was tested in RT-qPCR on the day of inoculation and day 7 for detection of virus replication.

A

	Original	Weighted	Simplified
Samples	2028	1849	1849
True negative	1816	1657.67	1657
True positive	90	86.83	89
False negative	120	102.5	101
False positive	2	2	2
Sensitivity	0.43	0.46	0.47
Specificity	0.99	0.99	0.99

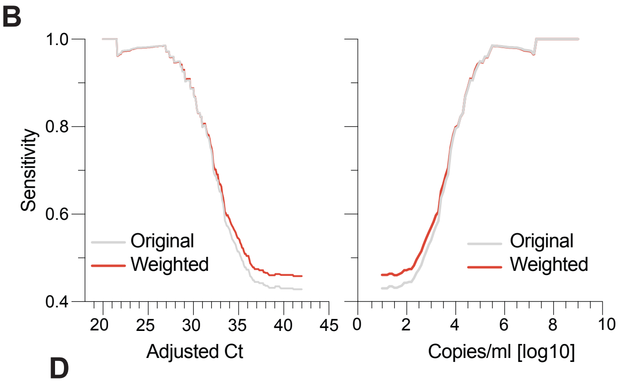

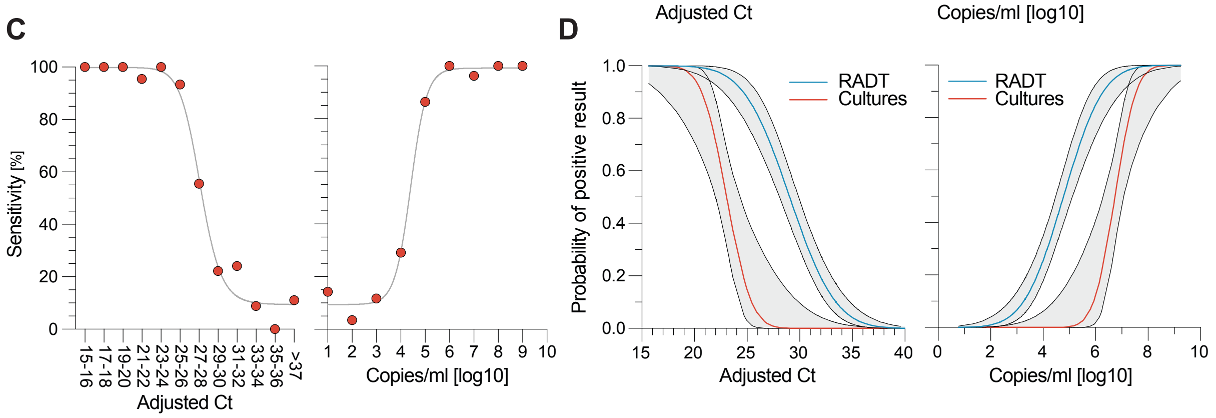

Fig. S2. Modified data analyses to correct for repeated testing of single individuals.
(A) Comparison of RADT performance data in either a weighted or simplified (only first swab result) fashion. **(B)** The cumulative sensitivity of the RADT is stratified by adjusted Ct values and RNA load in log copies/ml. **(C)** Sensitivity of RADT is stratified by adjusted Ct values and RNA load in log copies/ml for simplified analysis (only first swab result). **(D)** Probability of positive result for RADT and viral cultures in the context of adjusted Ct values and RNA load (Probit-Model, R-function GLMMadaptive). All weighted p-values remain $p < 0.0001$, Generalized Estimating Equations.
